# *Staphylococcus aureus*‐derived xanthine exacerbates allergic rhinitis‐induced olfactory dysfunction through promoting Th17 cell differentiation and IL‐17A production

**DOI:** 10.1002/cti2.70127

**Published:** 2026-11-01

**Authors:** Yue Hu, Wenjing Wang, Ting Yang, Xiaoyu Song, Yao Wang, Yuanchao Cheng, Yakui Mou, Chao Ren, Xicheng Song

**Affiliations:** ^1^ Department of Otorhinolaryngology, Head and Neck Surgery, Yantai Yuhuangding Hospital Qingdao University Yantai China; ^2^ Shandong Provincial Key Laboratory of Neuroimmune Interaction and Regulation Yantai China; ^3^ Shandong Provincial Clinical Research Center for Otorhinolaryngologic Diseases Yantai China; ^4^ Shandong Engineering Research Center for Precision Diagnosis and Treatment of Airway Diseases Yantai China; ^5^ Yantai Key Laboratory of Otorhinolaryngologic Diseases Yantai China; ^6^ Yantai Clinical Research Center for Otorhinolaryngologic Diseases Yantai China; ^7^ Center for Clinical Laboratory, Beijing Youan Hospital Capital Medical University Beijing China; ^8^ Department of Neurology, Yantai Yuhuangding Hospital Qingdao University Yantai China; ^9^ Yantai Municipal Key Medical and Health Laboratory of Yantai Yuhuangding Hospital (Interdisciplinary Brain Science and Geriatric Health Laboratory) Yantai China

**Keywords:** allergic rhinitis, IL‐17A, olfactory dysfunction, *Staphylococcus aureus*, xanthine

## Abstract

**Objectives:**

Allergic rhinitis (AR) is frequently accompanied by olfactory dysfunction (OD), yet the role of microbial metabolites in this process remains poorly elucidated. This study aimed to investigate how xanthine, a metabolite derived from *Staphylococcus aureus* (*Sa*), exacerbates AR‐induced OD by promoting Th17 cell differentiation and IL‐17A production.

**Methods:**

Multi‐omics analyses, comprising metabolomics, proteomics and 16S rRNA gene sequencing, were performed on AR patients and mouse models with AR. *Sa* was cultured and key metabolites in its supernatant were detected using untargeted metabolomics. AR mice were nasally challenged with *Sa* supernatant or xanthine, and comparisons were made with mice with chronic AR and OD. The therapeutic potential of an anti‐IL‐17A neutralising antibody was assessed in AR mice stimulated with *Sa* supernatant or xanthine.

**Results:**

Purine metabolism was significantly enriched, and xanthine levels were elevated in AR patients and mouse models, particularly those with OD. Airway microbial dysbiosis in AR mice was characterised by increased *Staphylococcus* abundance, and the *Sa* infection pathway was enriched in patients with AR. Untargeted metabolomics of *Sa* supernatant revealed increased xanthine levels and enrichment of purine metabolism. Nasal challenge with *Sa* supernatant or xanthine significantly exacerbated OD. Mechanistically, xanthine promoted Th17 cell differentiation and IL‐17A production. Notably, IL‐17A neutralisation effectively alleviated xanthine‐exacerbated OD, indicating a pivotal role for the IL‐17A pathway in this process.

**Conclusion:**

*Sa*‐derived xanthine aggravates AR‐induced OD by driving Th17 cell differentiation and IL‐17A production. These findings highlight the xanthine–IL‐17A pathway as a potential therapeutic target for improving olfactory function in AR.

## Graphical abstract

*Staphylococcus aureus*‐derived xanthine exacerbates allergic rhinitis‐associated olfactory dysfunction by promoting Th17 cell differentiation and IL‐17A production. IL‐17A neutralisation alleviates olfactory sensory neuron loss and improves olfactory function.

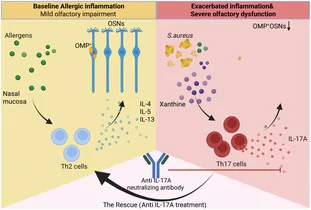

## Introduction

Allergic rhinitis (AR), an immunoglobulin (Ig)E‐mediated chronic inflammatory disorder of the upper respiratory tract, affects 10–40% of the global population and 20.9% of individuals in China, with steadily rising prevalence.^1^, ^2^ Beyond classic nasal symptoms, including nasal congestion, rhinorrhoea, sneezing and pruritus, olfactory dysfunction (OD) has emerged as a common yet long‐overlooked clinical manifestation of AR, increasingly garnering attention from clinicians and researchers.^3^, ^4^ Clinical evidence indicates that up to 47.1% of AR patients experience varying degrees of OD,^5^ which not only impairs quality of life but also predisposes patients to psychosocial disorders.^6^, ^7^ Despite this burden, the pathogenesis of AR‐induced OD remains incompletely elucidated, and effective targeted therapies are lacking.

Recent advances in microbiome research have opened a new avenue for understanding AR and its associated complications. Airway microbial dysbiosis is increasingly recognised as a key contributor to AR pathogenesis and OD, although relevant studies are limited.^8^, ^9^, ^10^, ^11^, ^12^ Current research primarily focuses on clinical cohort analyses. AR patients exhibit increased nasal abundance of *Moraxella*, *Haemophilus*, *Streptococcus* and *Staphylococcus*,^10^ and the nasal colonisation rate and abundance of *Staphylococcus aureus* (*Sa*) are positively correlated with AR severity.^12^, ^13^ A recent study further demonstrated that *Streptococcus salivarius* exacerbates AR‐related inflammation by binding to the nasal mucosal protein mucin 5AC.^9^ However, the specific role of the nasal microbiome in AR‐induced OD remains poorly defined, warranting further investigation of the functional contributions of dominant nasal bacteria and microbiome–host crosstalk.

Previous studies identified aberrant purine metabolism in AR patients compared with healthy subjects.^14^, ^15^ Notably, purine metabolism is the most significantly enriched pathway distinguishing chronic rhinosinusitis (CRS) patients with OD from those without OD, and this metabolic dysregulation is closely linked to nasal microbial alterations.^11^ Through metabolomic profiling of representative bacterial genera associated with AR,^16^ we identified xanthine as a central intermediate in purine metabolism and the most consistently upregulated shared bacterial metabolite. Xanthine levels are elevated in nasal lavage fluid (NLF) of AR patients,^15^ and *in vitro* studies have shown that xanthine promotes T helper 17 (Th17) cell differentiation via the endoplasmic reticulum stress pathway.^15^, ^17^ However, the precise role of this microbial metabolite in regulating the immune responses underlying AR‐induced OD, and the mechanisms involved, remain unclear.

In the present study, we integrated metabolomics data from multiple clinical cohorts of patients with chronic nasal diseases with findings from mouse models to investigate how *Sa*‐derived xanthine contributes to AR‐induced OD by promoting Th17 cell differentiation and interleukin (IL)‐17A production. Our findings extend the current understanding of microbiome‐metabolite‐host immune crosstalk in AR‐induced OD, highlighting a potential role for *Sa*‐associated xanthine in AR‐induced OD These results may clarify the pathogenic mechanisms of OD in AR and suggest potential biomarkers for disease susceptibility, as well as novel therapeutic strategies targeting microbial metabolism and immune pathways.

## Results

### Purine metabolism is dysregulated in patients with chronic nasal diseases and xanthine is elevated in AR patients and murine models

To detect shared metabolic alterations associated with chronic nasal inflammation, we integrated and compared metabolic pathway enrichment results reported in previous clinical metabolomic studies of AR and CRS.^11^, ^12^, ^15^, ^18^ These studies analysed NLF from children with AR, serum from adults with AR, nasal mucus from young and elderly patients with CRS, and nasal mucus from CRS patients with or without OD. Although AR and CRS represent distinct inflammatory disorders with different endotypes, both involve persistent mucosal inflammation. We identified purine metabolism as the only consistently enriched pathway. Further analysis revealed increases in several metabolites involved in purine metabolism, including xanthine, xanthosine, adenosine monophosphate (AMP), 3′‐AMP and uric acid, across these clinical cohorts (Figure 1a).

**Figure 1:**
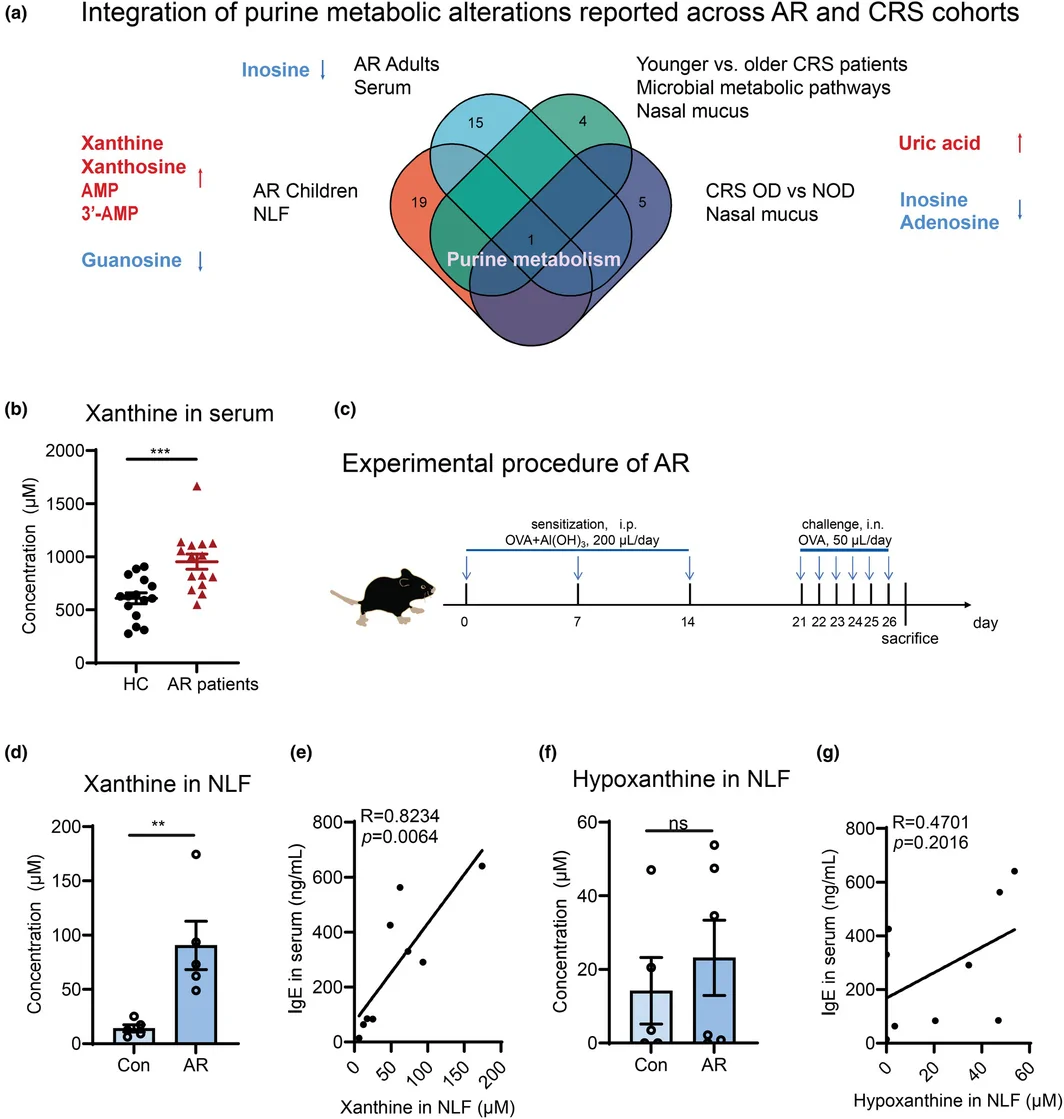
Purine metabolism was dysregulated in patients with chronic nasal diseases, with elevated xanthine levels in allergic rhinitis (AR) patients and murine models. **(a)** Integration of purine metabolic alterations reported across AR and chronic rhinosinusitis (CRS) cohorts. **(b)** Serum xanthine concentrations in patients with AR and healthy controls. **(c)** Experimental procedure for establishment of the AR mouse model. **(d, e)** Xanthine concentrations in nasal lavage fluid (NLF) and correlation between NLF xanthine concentrations and serum immunoglobulin (Ig)E levels. **(f, g)** Hypoxanthine concentrations in NLF and correlation between NLF hypoxanthine concentrations and serum IgE levels. Each symbol represents an individual mouse or human subject. Data are presented as the mean ± SEM (*n* = 3–5/group for mouse experiments). ns, not significant; **P < 0.01, ***P < 0.001.

Given the established roles of xanthine oxidase and xanthine in the progression of allergic and airway diseases, we first focused on xanthine. Serum concentrations of xanthine were significantly higher in AR patients than in HCs (Figure 1b), leading us to generate an OVA‐induced mouse model of AR (Figure 1c). Compared with the control group, the AR model mice showed significant increases in nasal rubbing frequency (Supplementary figure 1A), serum IgE concentration (Supplementary figure 1B) and the levels of Th2‐related cytokines IL‐4, IL‐5 and IL‐13 in NLF and nasal mucosa (Supplementary figure 1C–E), confirming successful establishment of the AR model. Histologically, AR mice exhibited a marked increase in inflammatory cell infiltration in the respiratory mucosa and thickening of the respiratory epithelium (Supplementary figure 1F). As expected, xanthine concentrations were significantly elevated in NLF (Figure 1d) and positively correlated with serum IgE levels (Figure 1e). In contrast, hypoxanthine concentrations in NLF did not differ significantly between groups and were not significantly correlated with IgE levels (Figure 1f and g).

### *Staphylococcus* is enriched in AR airways and *Sa*‐derived xanthine is a key purine metabolite

Previous studies have shown an association between dysregulated purine metabolism and microbiota dysbiosis,^19^, ^20^ and airway microbiota dysregulation has been confirmed in AR patients.^9^, ^21^ We therefore investigated whether disordered purine metabolism and xanthine elevation are driven by alterations in the airway microbiota. To explore such changes in AR, paired NLF and BALF samples were collected from control and AR mice and subjected to 16S rRNA gene amplicon sequencing (Figure 2a). Analysis of the Gut Microbiome Health Index (GMHI) and Microbiota Dysbiosis Index (MDI) in NLF and BALF revealed impaired microbiota health and more severe dysbiosis in AR mice (Supplementary figure 2A). At the genus level, only *Staphylococcus* showed a significant increase in both NLF and BALF in AR mice compared with that in the control group (Figure 2b, Supplementary figure 2B), consistent with previous findings of increased nasal carriage and abundance of *Sa* in AR patients.^13^, ^22^ Concurrently, the *Sa* infection pathway was significantly enriched in serum from AR patients compared with that from HCs (Figure 2c). Elevated IgG antibodies against inactivated *Sa* in the serum of AR patients and model mice further supported enhanced host immune recognition of *Sa*‐associated antigens (Figure 2d and e).

**Figure 2:**
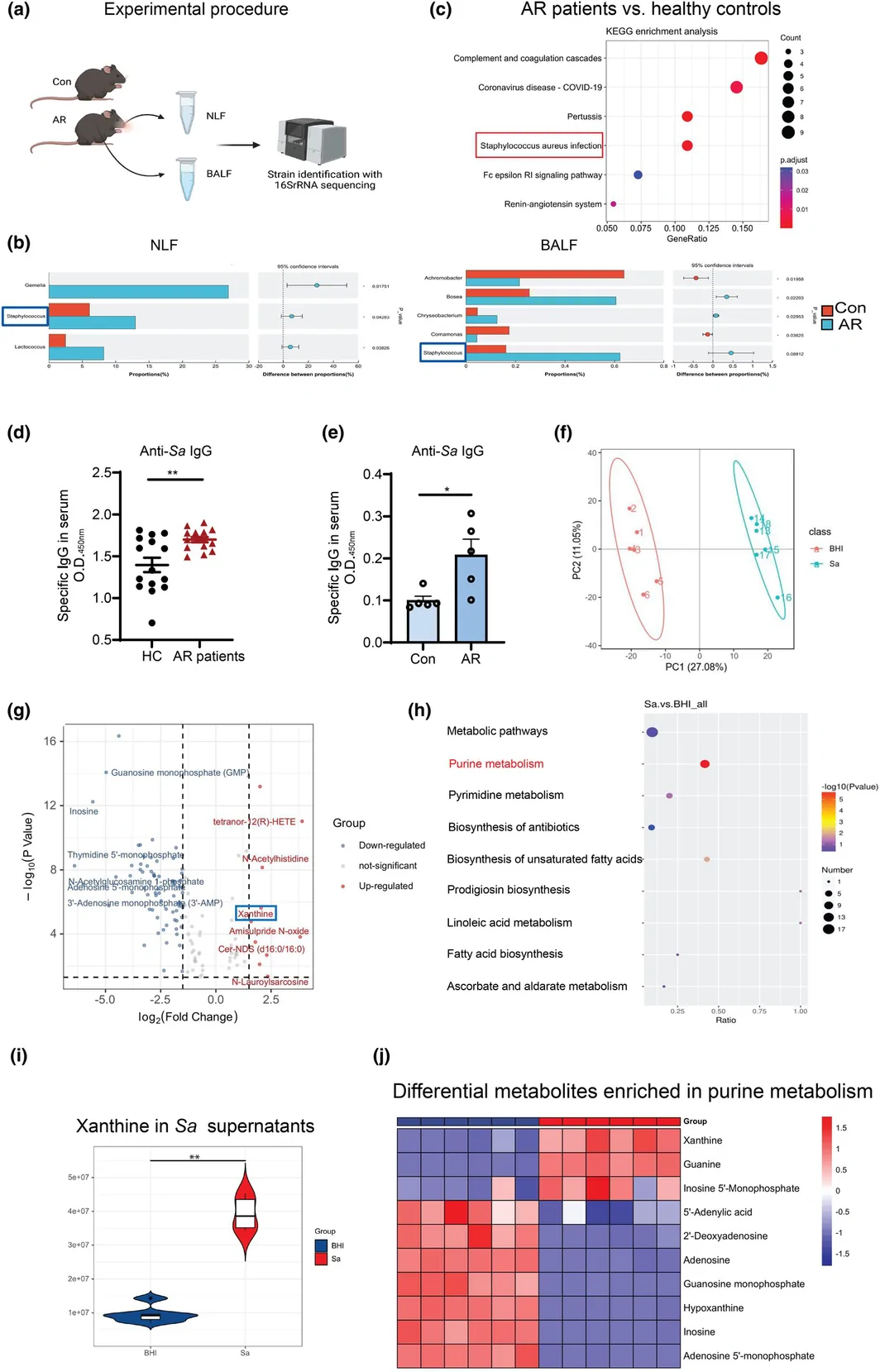
*Staphylococcus aureus* (*Sa*) was enriched in the airway microbiota of allergic rhinitis (AR), and xanthine was a prominent *Sa‐*derived purine metabolite. **(a)** Experimental procedure. Nasal lavage fluid (NLF) and bronchoalveolar lavage fluid (BALF) were collected and subjected to 16S rRNA gene amplicon sequencing. **(b)** Differentially abundant bacterial genera in NLF and BALF from control and AR mice. **(c)** Kyoto Encyclopedia of Genes and Genomes (KEGG) enrichment analysis of upregulated serum proteins in patients with AR compared with healthy controls. **(d, e)** Serum immunoglobulin (Ig)G antibodies against inactivated *Sa* in patients with AR **(d)** and AR mice **(e)**. **(f)** Principal component analysis (PCA) of metabolites detected in brain heart infusion (BHI) control medium and *Sa* culture supernatant. **(g)** Volcano plot of differential metabolites between BHI medium and *Sa* culture supernatant. **(h)** KEGG pathway enrichment of differential metabolites. **(i)** Relative abundance of xanthine in BHI medium and *Sa* supernatant. **(j)** Heatmap of metabolites enriched in purine metabolism. Each circle or triangle represents an individual mouse or subject. The data are expressed as the mean ± SEM (*n* = 3–15/group). *P < 0.05, **P < 0.01.

To explore the role of *Sa‐*derived metabolites in AR pathogenesis, we conducted untargeted metabolomics on *Sa* culture supernatant to identify metabolite components. PCA showed distinct differences between *Sa* supernatant and control medium (Figure 2f). A volcano plot indicated that xanthine was the most strongly upregulated metabolite in *Sa* supernatant (Figure 2g). Purine metabolism was the most significantly enriched Kyoto Encyclopedia of Genes and Genomes (KEGG) metabolic pathway (Figure 2h), and xanthine was the most highly upregulated metabolite within this pathway (Figure 2i and j). Collectively, these results identified xanthine as a prominent *Sa*‐derived metabolite *in vitro* and suggested that *Sa*‐associated xanthine may contribute to AR pathogenesis.

### Xanthine accumulates with AR progression and is associated with AR‐induced OD

Next, we generated a chronic AR mouse model based on chronic asthma models (Figure 3a). As expected, nasal rubbing frequency, serum IgE concentrations, and IL‐4, IL‐5 and IL‐13 concentrations in both NLF and serum were significantly elevated (Supplementary figure 3A–D). Notably, most chronic AR model mice exhibited OD (Figure 3b). The respiratory region of the nasal cavity in these mice showed increased respiratory epithelial thickness and goblet cell numbers, together with inflammatory cell infiltration (Supplementary figure 3E, F). Consistent with OD, olfactory epithelial thickness (Figure 3c) and the OMP‐positive area were significantly reduced, indicating loss or impairment of olfactory sensory neurons (OSNs) (Figure 3d). Untargeted metabolomics revealed that xanthine was the second most upregulated metabolite in the serum of chronic AR mice (Supplementary figure 3G, H). The relative abundance of xanthine increased with progression from acute to chronic AR (Figure 3e). Meanwhile, xanthine levels were also significantly elevated in the olfactory epithelium (Figure 3f). Xanthine levels in both serum and the olfactory epithelium were positively correlated with impaired olfactory function (Figure 3g and h). Consistent with clinical cohorts, purine metabolism was the most significantly enriched pathway when comparing acute and chronic AR mice (Figure 3i). Taken together, these results suggested that xanthine accumulation is associated with AR progression, particularly AR‐induced OD, prompting further investigation of the underlying mechanisms.

**Figure 3:**
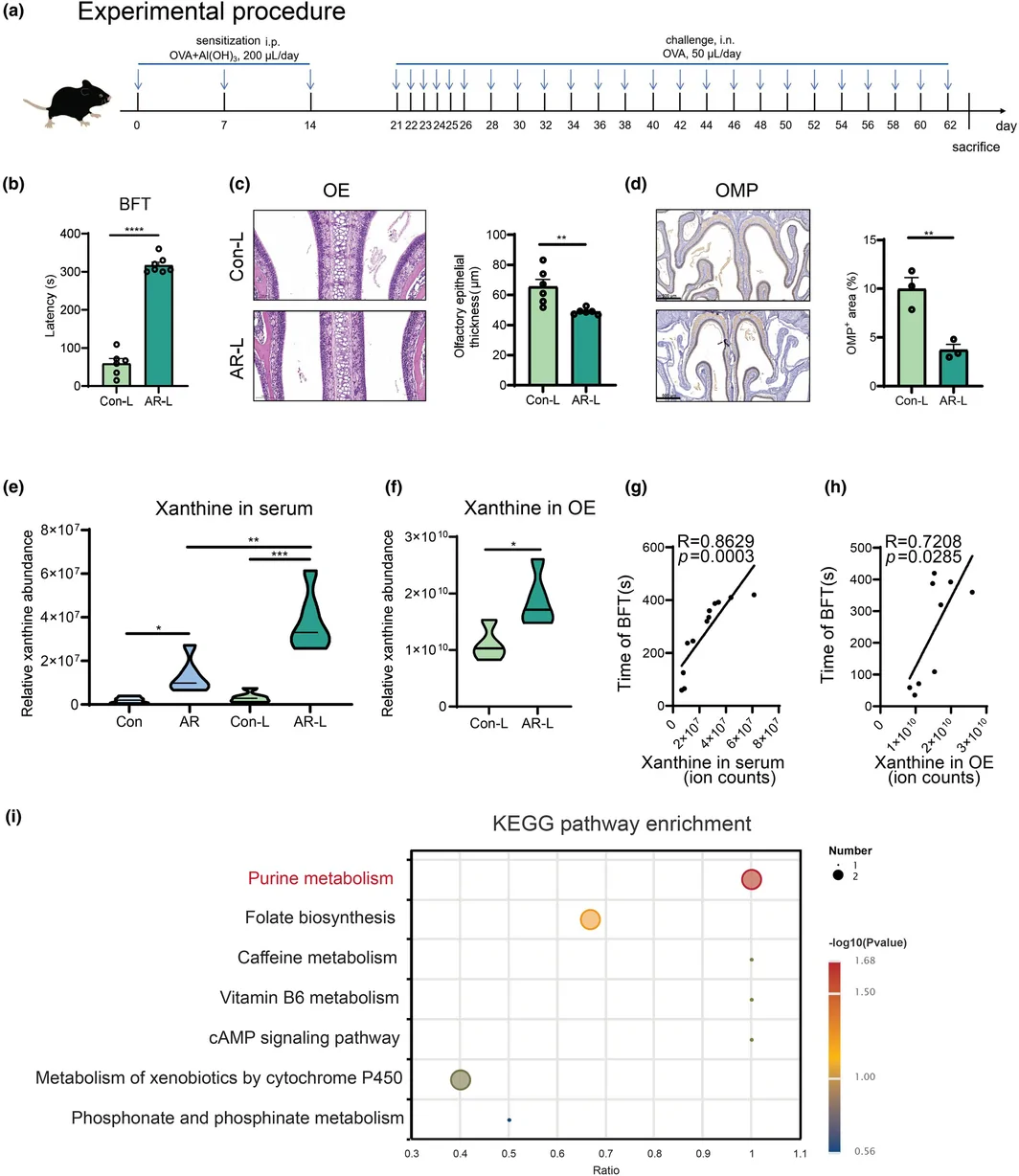
Xanthine correlates with allergic rhinitis (AR)‐induced olfactory dysfunction. **(a)** Experimental procedure establishing the chronic AR model. **(b)** Buried food test (BFT) latency. **(c)** Haematoxylin and eosin (H&E) staining of nasal olfactory region and quantification of olfactory epithelium thickness. **(d)** Immunohistochemical (IHC) staining of olfactory marker protein (OMP) and quantification of OMP‐positive area. **(e)** Relative abundance of xanthine in serum of mice in acute AR (AR) and chronic AR (AR‐L) groups. **(f)** Relative abundance of xanthine in the olfactory epithelium (OE) of AR‐L group mice. **(g, h)** Correlation analysis between xanthine levels in serum **(g)** and OE **(h)** and BFT latency. **(i)** Kyoto Encyclopedia of Genes and Genomes (KEGG) pathway enrichment of differential metabolites in AR‐L group. Each circle represents an individual mouse. The data are expressed as the mean ± SEM (*n* = 3–8/group). *P < 0.05, **P < 0.01, ***P < 0.001, ****P < 0.0001.

### *Sa*‐derived xanthine exacerbates AR and OD by promoting Th17 cell differentiation and IL‐17A production

Based on our findings in chronic AR mice and our previous study,^23^ we repeatedly stimulated AR mice with *Sa* supernatant or xanthine (Figure 4a). *Sa* supernatant and xanthine stimulation resulted in increased respiratory epithelial thickness and goblet cell numbers (Supplementary figure 4A, B) comparable to those in the chronic AR model, as well as reductions in olfactory epithelium thickness (Figure 4b and d) and OMP‐positive area (Figure 4c and e). Notably, although *Sa* supernatant and xanthine did not increase IL‐4, IL‐5 and IL‐13 levels in the NLF of AR mice (Supplementary figure 4C), IL‐17A was significantly elevated (Figure 4f), consistent with findings in chronic AR models. IL‐17A levels were also increased in the nasal epithelium, BALF and serum of AR‐L mice (Supplementary figure 4D). In AR patients, serum concentrations of IL‐17A were increased compared with HCs (Figure 4g). Stimulation with *Sa* supernatant or xanthine significantly increased the proportion of Th17 cells in mLNs (Figure 4h), whereas no significant increase was observed in the spleen (Supplementary figure 4E).

**Figure 4:**
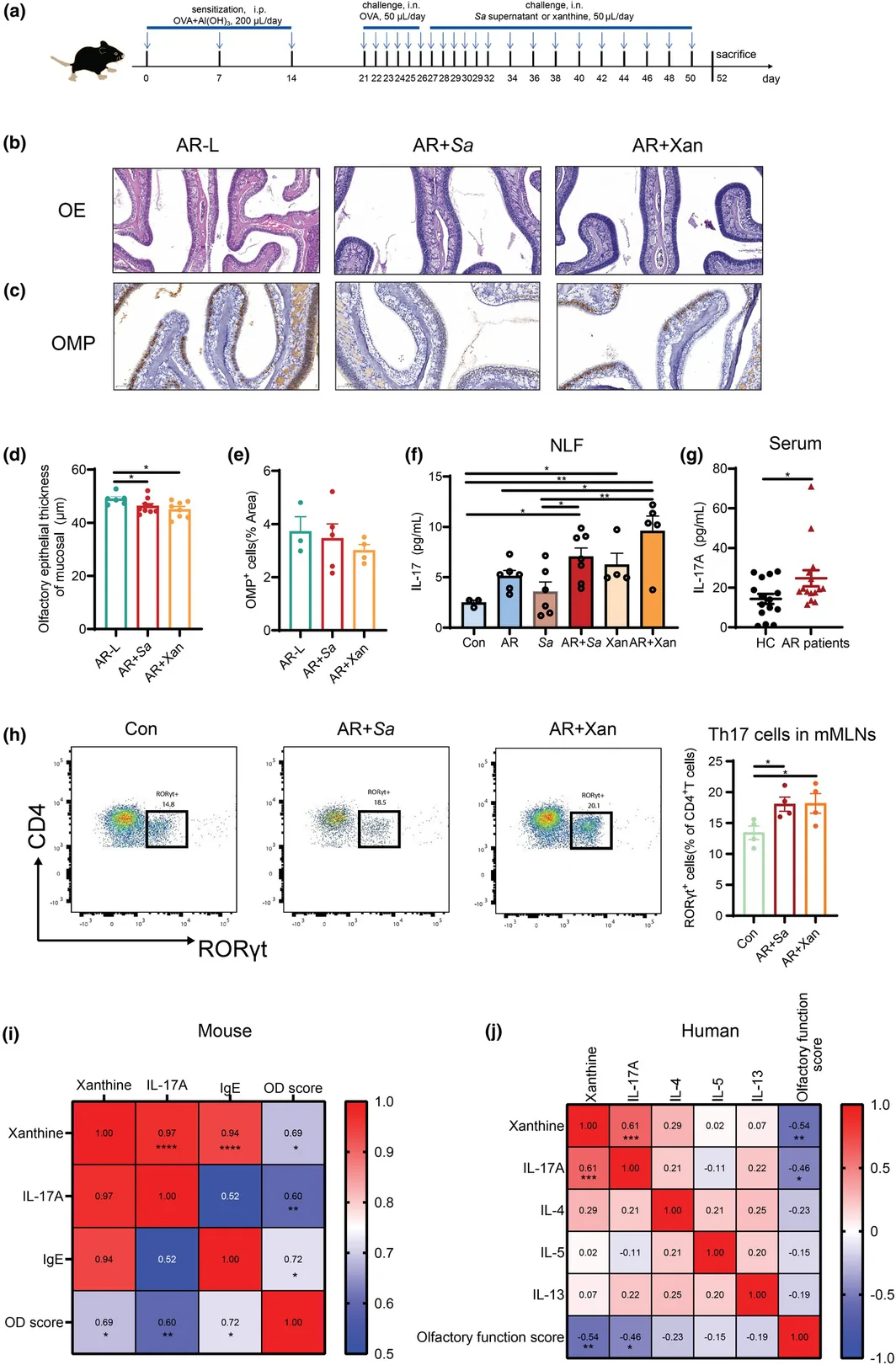
*Staphylococcus aureus* (*Sa*)‐derived xanthine exacerbates allergic rhinitis (AR) and olfactory dysfunction by promoting Th17 cell differentiation and IL‐17A production. **(a)** Experimental procedure for repeated stimulation of AR mice with *Sa* supernatant or xanthine. **(b, d)** Haematoxylin and eosin (H&E) staining of nasal olfactory region and quantification of olfactory epithelium thickness. **(c, e)** Immunohistochemical (IHC) staining of olfactory marker protein (OMP) and quantification of OMP‐positive area. **(f)** Concentrations of IL‐17A in nasal lavage fluid (NLF). **(g)** Concentrations of IL‐17A in serum of AR patients and healthy subjects. **(h)** Representative flow cytometry plots and quantification of the percentage of Th17 cells in mLNs. **(i)** Correlation matrix of NLF xanthine and IL‐17A concentrations, serum immunoglobulin (Ig)E concentrations and buried food test (BFT) latency in mice. **(j)** Correlation matrix of serum xanthine, IL‐17A, IL‐4, IL‐5 and IL‐13 concentrations and olfactory function scores in patients with AR and healthy controls (HCs). Each circle and triangle represents an individual mouse or subject. The data are expressed as the mean ± SEM (*n* = 3–15/group). ns, not significant; *P < 0.05, **P < 0.01.

Correlation analysis of NLF xanthine and IL‐17A concentrations, serum IgE concentrations and BFT latency in mice revealed significant positive correlations among xanthine, IL‐17A and BFT latency (Figure 4i). Xanthine was also positively correlated with IL‐17A in AR patients and HCs, but not with Th2 cytokines, and both were negatively correlated with olfactory function (Figure 4j). Proteomic analysis of all differentially expressed proteins and upregulated proteins in serum further identified enrichment of the Th17 cell differentiation pathway in AR patients compared with HCs (Supplementary figure 5). Subsequently, multiple serum IgEs were measured. *Sa* supernatant and xanthine induced increases in IgG, IgG1 and IgA, but not IgE (Supplementary figure 4F). These results suggested that *Sa*‐derived xanthine exacerbates AR and AR‐induced OD in association with enhanced Th17 cell differentiation and IL‐17A production.

### IL‐17A neutralisation attenuates AR and OD induced by *Sa*‐derived xanthine

To determine whether *Sa*‐derived xanthine exacerbates AR via the IL‐17A pathway, we performed an *in vivo* rescue experiment. An anti‐IL‐17A neutralising antibody (αIL‐17A) was intranasally administered to AR mice stimulated with either *Sa* supernatant or xanthine, as well as to chronic AR mice (Figure 5a). OD induced by *Sa* supernatant or xanthine was comparable to that observed in chronic AR mice, and IL‐17A neutralisation significantly alleviated OD (Figure 5b). Serum levels of IgG and IgG1 were also decreased (Figure 5c), together with reduced OVA‐specific IgE levels (Figure 5d).

**Figure 5:**
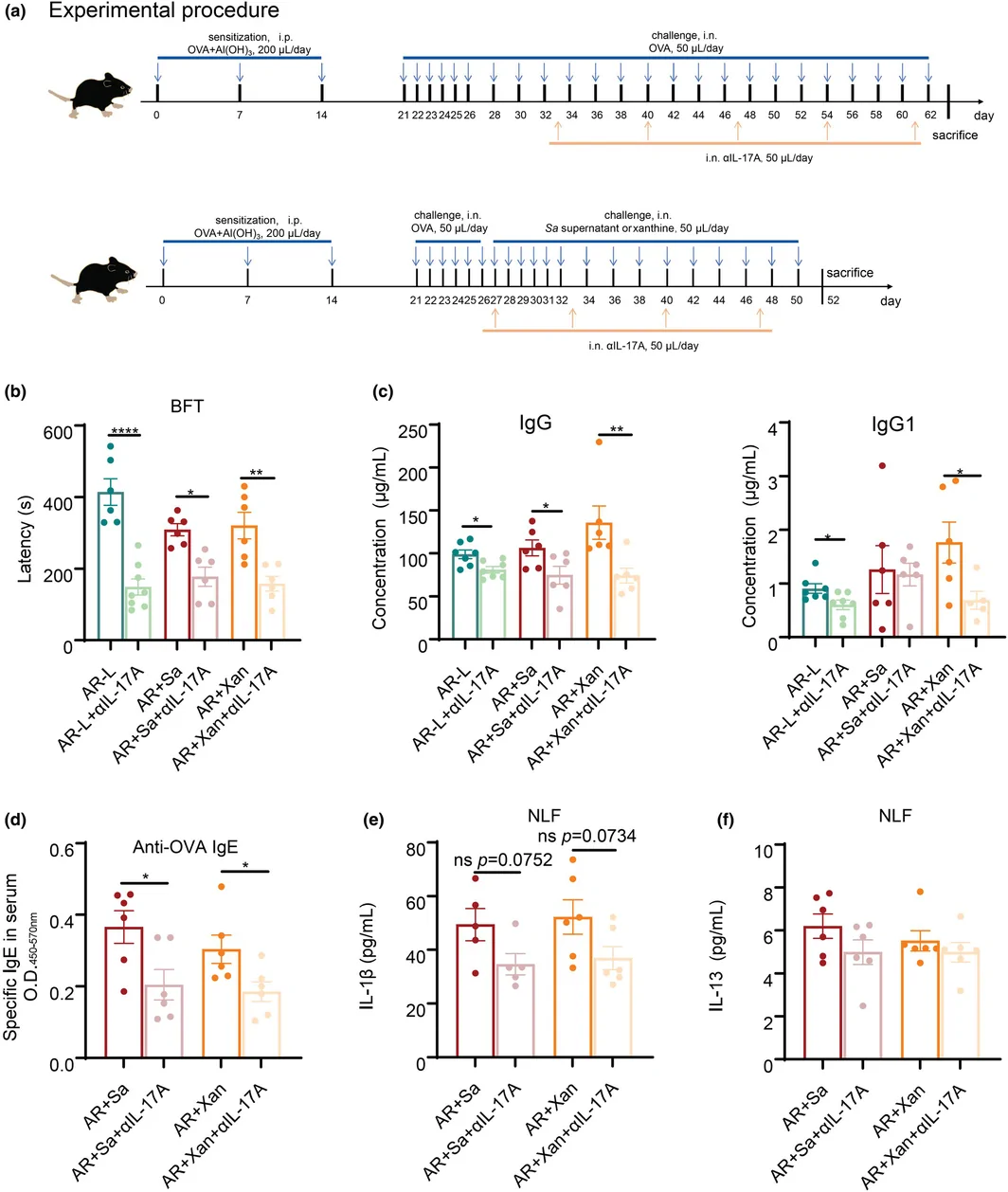
IL‐17A neutralisation attenuates allergic rhinitis (AR) and olfactory dysfunction induced by *Staphylococcus aureus* (*Sa*)‐derived xanthine. **(a)** Experimental procedure for IL‐17A neutralisation in chronic AR mice and acute AR mice stimulated with *Sa* supernatant or xanthine. AR‐L represents the chronic AR mice; AR + *Sa* mice received intranasal administration of *Sa* culture supernatant, whereas AR + Xan mice were challenged with xanthine. αIL‐17A represents intranasal treatment with an anti‐IL‐17A antibody. **(b)** Buried food test (BFT) latency. **(c)** Serum concentrations of immunoglobulin (Ig)G and IgG1. **(d)** Levels of specific IgE antibodies against ovalbumin(OVA) in serum. **(e, f)** Concentrations of IL‐1β **(e)** and IL‐13 **(f)** in nasal lavage fluid (NLF). Each circle represents an individual mouse. Data are expressed as the mean ± SEM (*n* = 5–8/group). ns, not significant; *P < 0.05, **P < 0.01, ****P < 0.0001.

NLF IL‐1β levels showed a decreasing trend following IL‐17A neutralisation, although the differences did not reach statistical significance (Figure 5e), whereas NLF IL‐13 levels remained unchanged (Figure 5f). In addition, we found that BFT latency was significantly correlated with several anxiety‐related behavioural parameters, and intranasal IL‐17A neutralisation significantly attenuated anxiety‐like behaviours in chronic AR mice (Supplementary figure 6). Taken together, these rescue experiments support a key role for IL‐17A in the pathological effects of xanthine, and are consistent with our hypothesis that *Sa*‐derived xanthine exacerbates AR and OD, at least in part, through IL‐17A.

## Discussion

The mechanism underlying OD in AR remains incompletely understood. Recent clinical studies have linked the nasal microbiome to OD and cognitive decline,^8^, ^24^, ^25^ yet few studies have focused on AR‐induced OD. In the present study, purine metabolism was dysregulated and xanthine levels were elevated in clinical cohorts of patients with nasal diseases and in AR model mice, particularly those with OD. *Staphylococcus* abundance was increased in the airways of AR model mice, while AR patients exhibited elevated circulating xanthine levels and enrichment of the *Sa* infection pathway. Untargeted metabolomics further identified xanthine as a prominent metabolite in *Sa* culture supernatant. Further exploration showed that xanthine promotes Th17 cell differentiation and IL‐17A production and exacerbates AR‐induced olfactory epithelial damage and OD.

Dysfunction of the airway microbiome is associated with the development of AR. Previous studies have reported increased nasal abundance and carriage of *Sa* in patients with AR and allergic diseases,^13^, ^22^, ^26^, ^27^ but the mechanism remains poorly understood. In addition to classic superantigens and toxins, we previously showed that *Sa* lysate induced an IgE response via memory B cells in nasal polyps.^28^ In this study, we found that *Staphylococcus* was the only taxon with significantly increased abundance in both the NLF and BALF of AR mice. Based on these findings, we selected *Sa* for further investigation. The enrichment of the *Sa* infection pathway in AR patient serum and elevated anti‐*Sa* IgG antibodies in patients with AR and model mice supported enhanced host immune recognition of *Sa*‐associated components, but did not establish increased bacterial burden, colonisation or active infection. Recent studies have demonstrated altered nasal microbial composition and *Sa*‐associated dysbiosis in AR, suggesting that chronic allergic inflammation may reshape host–microbial interactions and enhance immune responses to microbial antigens. However, we acknowledge that direct quantification of nasal *Sa* abundance in AR patients was not performed in this study. Future studies incorporating bacterial culture or microbiome sequencing will be required to determine whether altered antibody responses are associated with changes in microbial colonisation. Importantly, although *Sa* superantigens are well‐established drivers of type 2 inflammation, our functional experiments indicated that *Sa*‐associated products, particularly xanthine‐containing *Sa* culture supernatant, can modulate host immunity, suggesting that microbially derived metabolites may contribute to disease progression beyond changes in bacterial abundance or exposure to bacterial components.

Xanthine, a key metabolite in purine metabolism, is catalysed by xanthine oxidase to form uric acid, the end product of purine metabolism.^29^, ^30^ Purine metabolism and xanthine have been implicated in the development of allergic and chronic respiratory diseases^15^, ^31^, ^32^, ^33^, ^34^; however, most studies have focused on asthma and CRS and have lacked mechanistic exploration. Xu *et al*. recently reported that abnormal purine metabolism in nasal epithelial cells modulates AR by regulating the balance between Th17 and regulatory T cells,^15^ partly consistent with our findings. Notably, our study extends these findings by focusing on xanthine, a prominent metabolite identified in *Sa* culture supernatant, and explores its role in AR‐induced OD from the perspective of microbe–metabolite–host immune crosstalk. Although circulating xanthine‐associated metabolic alterations were observed, immune activation was not uniformly distributed across lymphoid organs, highlighting the importance of tissue‐specific inflammatory niches in determining T‐cell responses. These findings suggest that xanthine may serve as a metabolic indicator associated with olfactory impairment in AR. Future studies involving larger clinical cohorts and longitudinal analyses are required to determine its predictive value and clinical applicability.

Few studies have examined the immunoregulatory effects of xanthine, and available evidence regarding T‐cell responses remains limited. *In vitro* studies have shown that xanthine treatment promotes Th17 cell differentiation and affects the endoplasmic reticulum stress‐related pathway and ATF4.^15^, ^17^ However, whether this mechanism contributes to xanthine‐mediated Th17 responses in AR *in vivo* remains unclear. Our experimental data support the involvement of Th17‐related immune activation, while the upstream molecular events require further investigation. In established allergic inflammation, microbially derived metabolic signals may not necessarily augment the classical Th2–IgE axis but can introduce additional inflammatory dimensions, such as Th17–IL‐17A responses, thereby contributing to disease persistence and severity. Additionally, IL‐17A neutralisation significantly alleviated AR‐induced OD. Interestingly, a recent study showed that IL‐4, but not IL‐13, induces loss of smell in CRS,^35^ despite the close association between IL‐33 and OD in CRS,^36^ suggesting that OD may be triggered by multiple factors involving complex mechanisms. Our previous study identified the proinflammatory cytokine IL‐6 as a mediator of OD in AR.^37^ Recently, another study showed that IL‐17A contributes to age‐related OD,^38^ and our findings suggest that IL‐17A may represent an additional inflammatory mediator specifically involved in AR‐associated OD. Therefore, combined targeting of classic type 2 inflammation and the Th17/IL‐17A axis may provide a potential strategy for refractory AR with persistent OD. The present study had several limitations. First, although we integrated metabolomic findings from previous clinical cohort studies, our mechanistic investigations were primarily performed in animal models. Moreover, olfactory function in the clinical cohort was assessed using a subjective VAS, without objective psychophysical olfactory testing. Additional clinical studies incorporating longitudinal nasal microbiome profiling, microbial metabolomics and standardised psychophysical olfactory testing are warranted to determine whether *Sa*‐associated microbial alterations and metabolic changes are associated with OD in AR patients and to further validate the clinical relevance of this microbial–metabolic axis. Second, although xanthine can be generated through host purine metabolism, our findings suggest that *Sa*‐associated products may provide an additional metabolic source. Thus, microbially derived xanthine should be considered as a contributor rather than the exclusive source of increased xanthine during AR progression. Future studies using isotope‐labelling approaches, genetically modified bacterial strains and clinical nasal *Sa* isolates will be required to further elucidate the contribution of *Sa*‐derived xanthine. Third, although xanthine levels were validated by LC–MS/MS, the downstream metabolic regulation of xanthine requires further investigation. Uric acid in NLF was below the detection limit, whereas systemic uric acid showed an increasing trend following exogenous xanthine administration, suggesting that downstream xanthine metabolism remains functional *in vivo* (Supplementary figure 3I). Comprehensive metabolic tracing will be needed to fully characterise purine metabolic flux. Finally, the tissue‐specific regulation of xanthine‐induced immune responses remains unclear. The increased proportion of Th17 cells observed in mLNs, but not in the spleen, suggests that Th17 responses may differ across lymphoid compartments. However, the mechanisms underlying this difference were not investigated in the present study and warrant further investigation.

The discovery of the *Sa*–xanthine–IL‐17A axis provides a rationale for novel targeted therapies, particularly in severe AR patients with OD. One potential translational approach is to target the downstream immune effector through IL‐17A blockade. Secukinumab, a fully human neutralising monoclonal antibody against IL‐17A, has demonstrated clinical efficacy in Th17‐mediated diseases, such as psoriasis and ankylosing spondylitis.^39^, ^40^ These observations raise the possibility that IL‐17A‐targeted therapy may provide complementary benefit in Th17‐skewed AR, particularly in patients with persistent inflammation or OD that is inadequately controlled by conventional Th2‐directed therapies. However, the systemic blockade of IL‐17A may impair mucocutaneous host defences and increase susceptibility to bacterial and fungal infections, such as candidiasis.^41^ Therefore, whether localised modulation of IL‐17A signalling in the nasal mucosa could provide therapeutic benefit while limiting systemic adverse effects warrants further investigation.

Targeting the upstream xanthine metabolic pathway represents another potential therapeutic strategy, although it poses distinct pharmacological challenges. Allopurinol, a classic xanthine oxidase (XO) inhibitor, has shown anti‐inflammatory effects in preclinical models of asthma.^32^ However, XO participates in both the conversion of hypoxanthine to xanthine and the subsequent oxidation of xanthine to uric acid,^42^ indicating that systemic inhibition of this pathway may alter purine metabolic flux in a complex manner. In addition, systemic allopurinol can be associated with severe cutaneous adverse reactions (SCARs).^43^ These considerations suggest that broad systemic manipulation of purine metabolism may not be ideal for AR, and more selective approaches aimed at modulating xanthine‐associated signalling or local purine metabolism may be preferable. Finally, although direct clinical evidence is currently lacking, modulation of nasal microbial dysbiosis, particularly *Sa*‐associated metabolic activity, may represent a potential complementary strategy for controlling AR‐related inflammation and OD.

In conclusion, this study demonstrates that *Sa*‐derived xanthine can exacerbate AR‐induced OD by promoting Th17 cell differentiation and IL‐17A production. Our findings identify a mechanistic link between a bacterial metabolite and dysregulated type 17 immunity in AR‐associated OD and may provide new insights into targeting microbial metabolism and IL‐17A as potential therapeutic strategies for AR‐associated OD.

## Methods

### Clinical samples

Forty‐two native Han Chinese participants, comprising 22 AR patients and 20 healthy controls (HCs), were enrolled at Yantai Yuhuangding Hospital in a study approved by the hospital's Ethics Committee (approval no.: 2024‐574). Detailed inclusion and exclusion criteria for both the AR patients and HCs are provided in the Supplementary Materials. Following the clinical assessments, fasting peripheral blood was collected from each participant using standardised clinical protocols. The blood samples were promptly centrifuged, aliquoted into sterile cryovials and immediately stored at −80°C for further analysis. Subjective olfactory function was assessed using a standard 10‐cm visual analogue scale (VAS) in accordance with the European Position Paper on Rhinosinusitis and Nasal Polyps guidelines. Patients were asked to rate their olfactory function on a continuous VAS ranging from 0 (complete loss of smell) to 10 (normal sense of smell), with lower scores indicating greater olfactory impairment.

### Mice

Wild‐type (WT) male C57BL/6J mice (age: 8–10 weeks) were purchased from Vital River Laboratory (Beijing, China) and housed under specific pathogen‐free (SPF) conditions in individually ventilated cages at the animal facility of Yantai Yuhuangding Hospital. All experimental groups were housed in the same animal room under identical environmental conditions to minimise potential cage‐ or facility‐related microbiological variations. Autoclaved bedding and sterilised food and water were provided throughout the experimental period. All experiments were performed in strict accordance with protocols approved by the Animal Ethics Committees of Yantai Yuhuangding Hospital and Qingdao University.

### Preparation of *Sa* supernatant

*Sa* (ATCC 29213) was cultivated in 200 mL brain heart infusion (BHI) medium to an optical density at 600 nm of approximately 1.0 and then centrifuged at 12 000 rpm for 20 min at 4°C. The supernatant was collected, passed through 0.22‐μm filters under aseptic conditions and stored at −80°C until use.

### Establishment of AR mouse models

Mice were randomly assigned to control and AR groups. For sensitisation, mice in the AR group were intraperitoneally injected with a 200‐μL suspension containing ovalbumin (OVA) and Al(OH)_3_ on Days 0, 7 and 14, followed by daily intranasal challenge with 50 μL OVA solution for 6 consecutive days. To establish the chronic AR model (AR‐L group), mice were further challenged every other day for 5 weeks, from Days 28 to 62. To explore the role of *Sa*‐derived metabolites in AR pathogenesis, AR mice received intranasal administration of *Sa* culture‐derived supernatant (50 μL; AR + *Sa* group) or xanthine (50 μL of 250 μM xanthine; AR + Xan group) daily for 6 days, followed by alternate‐day challenges from Days 8 to 25 based on our previous workflow.^23^

### IL‐17A neutralisation experiment

Mice in the AR‐L group were intranasally challenged with an anti‐IL‐17A neutralising antibody (1.5 μg per mouse in 50 μL; Selleck Chemicals, TX, USA) on Days 33, 40, 47, 54 and 61. Mice in the AR + *Sa* and AR + Xan groups received the same treatment on Days 27, 33, 40 and 47.

### Collection of NLF and bronchoalveolar lavage fluid

Mice were anesthetised with 2% sodium pentobarbital, followed by tracheotomy. bronchoalveolar lavage fluid (BALF) was obtained by lavage of the lower airways twice with 0.8 mL phosphate‐buffered saline (PBS). NLF was then obtained separately by posterior nasal lavage, allowing the lavage fluid to pass through the nasal cavity and exit through the external nares. The nasal cavity was lavaged three times with 0.5 mL PBS. BALF and NLF samples were centrifuged at 4°C and 1500 rpm for 10 min, and the supernatants were harvested for subsequent analyses.

### Evaluation of nasal rubbing behaviour and the buried food test (BFT)

After the final nasal challenge with OVA or PBS, the number of nasal rubbing events in each mouse was recorded over a 5‐min period. Following a 12‐h fast, mice were placed in a test cage with 5 cm of bedding, with food buried at a random location 0.5 cm below the surface. The time required to locate the food was recorded. A maximum cut‐off time of 5 min was used for mice that failed to locate the buried food. For each test, fresh bedding and food were used, and each mouse was tested three times, with the mean value calculated.

### Xanthine detection

Xanthine concentrations in NLF were measured using liquid chromatography–tandem mass spectrometry (LC–MS/MS). Serum xanthine concentrations were determined using a commercial assay kit (Beyotime, Shanghai, China), in accordance with the manufacturer's instructions. Absorbance at 450 nm was measured using a SpectraMax i3x Multimode Plate Reader (Molecular Devices, CA, USA).

### Enzyme‐linked immunosorbent assay (ELISA)

Concentrations of IL‐4, IL‐5, IL‐13 and IL‐17A in NLF, serum and nasal mucosal proteins, as well as total serum IgE, IgG, IgG1 and IgA, were measured using commercial ELISA kits (Invitrogen, CA, USA), in accordance with the manufacturer's protocols. For the detection of serum OVA‐specific IgE antibodies, ELISA plates were coated with 100‐μL aliquots of OVA solution (20 μg/mL) overnight at 4°C. After washing, 100 μL aliquots of diluted serum (1:10) were added to each well and incubated for 2 h at room temperature. Peroxidase‐conjugated goat anti‐mouse IgE (H + L) (1:4000; SouthernBiotech, AL, USA) was used as the detection antibody. Subsequent steps were performed in accordance with the manufacturer's instructions, and absorbance was measured at 450 nm using a multimode plate reader.

### Histological staining

Nasal tissues were collected from each mouse, fixed in 10% formalin at room temperature for 24 h and then immersed in decalcification solution for 2 weeks. Nasal tissue sections (4‐μm thickness) were prepared and stained with haematoxylin and eosin (H&E) and periodic acid–Schiff (PAS) reagent kits. Images were captured using a Pannoramic Scan Digital Pathology System (3DHISTECH, Budapest, Hungary).

### Flow cytometric analysis of Th17 cells

Cellular suspensions from mouse mediastinal lymph nodes (mLNs) and spleens were prepared for flow cytometric analysis. Briefly, tissue samples were ground and filtered sequentially through 70‐ and 40‐μm cell strainers (BD Falcon, Bedford, MA, USA). After depletion of erythrocytes, cells were incubated with Fixable Viability Stain 510 for 10 min at room temperature in the dark to exclude dead cells. An Fc receptor blocker (anti‐mouse CD16/CD32; BD Pharmingen, San Diego, CA, USA) was added for 10 min to block non‐specific binding. Cells were then stained with allophycocyanin‐cyanine 7 (APC‐Cy7)‐conjugated anti‐CD3e (eBioscience, San Diego, CA, USA) and fluorescein isothiocyanate (FITC)‐conjugated anti‐CD4 (eBioscience) antibodies at room temperature for 30 min in the dark for surface marker detection. After washing and centrifugation, cells were incubated with a fixation and permeabilisation solution in accordance with the manufacturer's instructions. Following permeabilisation, cells were stained with phycoerythrin (PE)‐conjugated anti‐retinoic acid receptor‐related orphan receptor γT (RORγT) antibody (BD Pharmingen) at room temperature for 30 min in the dark for intracellular transcription factor detection. The results were analysed using a LSRFortessa X‐20 flow cytometer (BD Pharmingen) and the FlowJo software (Tree Star, Ashland, OR, USA).

### Immunohistochemistry and immunofluorescence staining

Paraffin sections were prepared for immunohistochemistry and immunofluorescence staining. Briefly, sections were incubated overnight at 4°C with primary rabbit antibodies against olfactory marker protein (OMP; 1:1000; Abcam, Cambridge, UK). After washing, the sections were incubated with a peroxidase‐conjugated goat anti‐rabbit secondary antibody (1:400; Invitrogen). Representative images were acquired using a Pannoramic Scan Digital Pathology System (3DHISTECH).

### Untargeted metabolomics analysis

Ultra‐high‐performance LC–MS/MS (UHPLC–MS/MS) was employed to analyse metabolites in *Sa* culture supernatant and mouse serum using a Vanquish UHPLC system (Thermo Fisher, Germany) coupled with an Orbitrap Q Exactive™ HF‐X mass spectrometer (Thermo Fisher). Samples were injected onto a Hypersil Gold column (100 × 2.1 mm, 1.9 μm) with a 17‐min linear gradient at a flow rate of 0.2 mL/min. The mobile phases were eluent A (0.1% formic acid in water) and eluent B (methanol) for positive ion mode, and eluent A (5 mM ammonium acetate, pH 9.0) and eluent B (methanol) for negative ion mode. The mass spectrometer was operated in positive and negative ion modes with a spray voltage of 3.5 kV, capillary temperature of 320°C, sheath gas flow rate of 35 psi, auxiliary gas flow rate of 10 L/min, S‐lens radiofrequency level of 60, and an auxiliary gas heater temperature of 350°C.

The raw data files generated by UHPLC–MS/MS were processed using Compound Discoverer 3.1 (Thermo Fisher) for peak alignment, peak picking and quantitation of each metabolite. Peak intensities were normalised to the total spectral intensity. Normalised data were used to predict the molecular formulas based on adduct ions, molecular ion peaks and fragment ions. Peaks were then matched against the mzCloud (https://www.mzcloud.org/), mzVault and MassList databases to generate accurate qualitative and relative quantitative results. Metabolites with a variable importance in projection (VIP) score > 1, a *P*‐value < 0.05 and a fold change ≥ 2 or ≤ 0.5 were considered differentially abundant. Principal components analysis (PCA), volcano plot construction and pathway enrichment analysis were performed using the R software (v3.4.3).

### Elevated plus‐maze (EPM) test

The elevated plus‐maze (EPM) apparatus consisted of a plus‐shaped platform elevated 50 cm above the floor, with two pairs of opposing arms: two open arms (30 cm × 6 cm) and two enclosed arms (30 cm × 6 cm × 20 cm). At the start of each trial, a mouse was placed on the central platform, facing an open arm, and allowed to explore freely for 5 min. The time spent in the open arms and the number of open‐arm entries were recorded and analysed.

### Open‐field test (OFT)

The open‐field arena was a square chamber (50 cm length × 50 cm width × 35 cm height) constructed from a medical‐grade polyvinyl chloride sheet (NJKEWBIO, Nanjing, China). For each test, a mouse was gently placed in the centre of the arena and permitted to explore freely for 5 min. The arena was virtually divided into a central zone (20 cm × 20 cm) and a peripheral zone. The time spent in the central zone and the number of entries into the central zone were recorded and analysed.

### Statistical analysis

Statistical analysis was performed using the GraphPad Prism 10.4 software (GraphPad Software, USA) and the R software (v3.4.3). Correlations between continuous variables were assessed using Pearson's correlation analysis. Student's *t*‐test was used to compare two groups, and one‐way analysis of variance was used to compare three or more treatment groups. All data are presented as the mean ± standard error of the mean (SEM), with a *P*‐value <0.05 considered statistically significant.

## Author contributions

**Yue Hu:** Conceptualization; methodology; software; investigation; funding acquisition; writing – original draft; writing – review and editing; visualization. **Wenjing Wang:** Methodology; resources. **Ting Yang:** Methodology; data curation. **Xiaoyu Song:** Methodology; data curation. **Yao Wang:** Methodology; data curation. **Yuanchao Cheng:** Methodology; data curation. **Yakui Mou:** Resources; project administration. **Chao Ren:** Funding acquisition; resources; project administration; writing – review and editing. **Xicheng Song:** Writing – review and editing; funding acquisition; project administration; resources.

## Conflict of interest

The authors declare no conflict of interest.

## Declaration of generative AI in scientific writing

AI was not used in scientific writing.

## Supporting information


Supplementary data 1

Supplementary figure 1

Supplementary figure 2

Supplementary figure 3

Supplementary figure 4

Supplementary figure 5

Supplementary figure 6

Supplementary table 1

## Data Availability

The datasets generated and analysed during the current study are not publicly available but can be obtained from Yue Hu upon reasonable request.
