# Supplementary material for: Staphylococcus aureus‐derived xanthine exacerbates allergic rhinitis‐induced olfactory dysfunction through promoting Th17 cell differentiation and IL‐17A production
Source: Clin Transl Immunology. 2026 Nov 1;15(11):e70127. doi: 10.1002/cti2.70127 (PMC13724795; doi:10.1002/cti2.70127)
Supplement: Supplementary file 1 [file CTI2-15-e70127-s002.pdf]

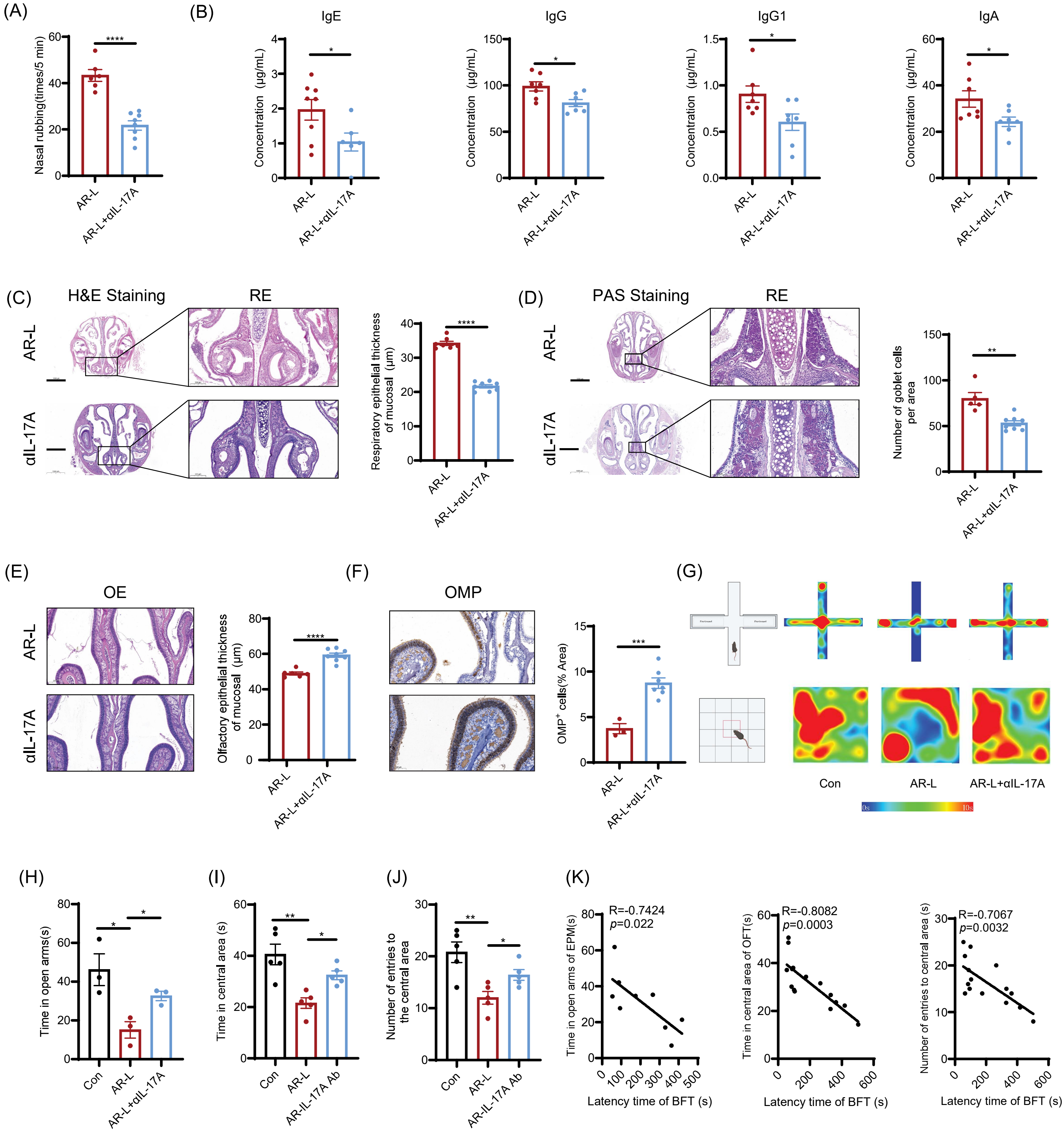

(A)
Nasal rubbing(times/5 min)
AR-L
AR-L+αIL-17A
(B)
IgE
IgG
IgG1
IgA
Concentration (μg/mL)
(C)
H&E Staining
RE
AR-L
αIL-17A
Respiratory epithelial thickness of mucosal (μm)
(D)
PAS Staining
RE
Number of goblet cells per area
(E)
OE
Olfactory epithelial thickness of mucosal (μm)
(F)
OMP
OMP+ cells(% Area)
(G)
Con
AR-L
AR-L+αIL-17A
0s
10s
(H)
Time in open arms(s)
(I)
Time in central area (s)
AR-IL-17A Ab
(J)
Number of entries to the central area
(K)
R=-0.7424
p=0.022
Time in open arms of EPM(s)
Latency time of BFT (s)
R=-0.8082
p=0.0003
Time in central area of OFT(s)
R=-0.7067
p=0.0032
Number of entries to central area (s)
