# Supplementary material for: Staphylococcus aureus‐derived xanthine exacerbates allergic rhinitis‐induced olfactory dysfunction through promoting Th17 cell differentiation and IL‐17A production
Source: Clin Transl Immunology. 2026 Nov 1;15(11):e70127. doi: 10.1002/cti2.70127 (PMC13724795; doi:10.1002/cti2.70127)
Supplement: Supplementary file 2 [file CTI2-15-e70127-s003.pdf]

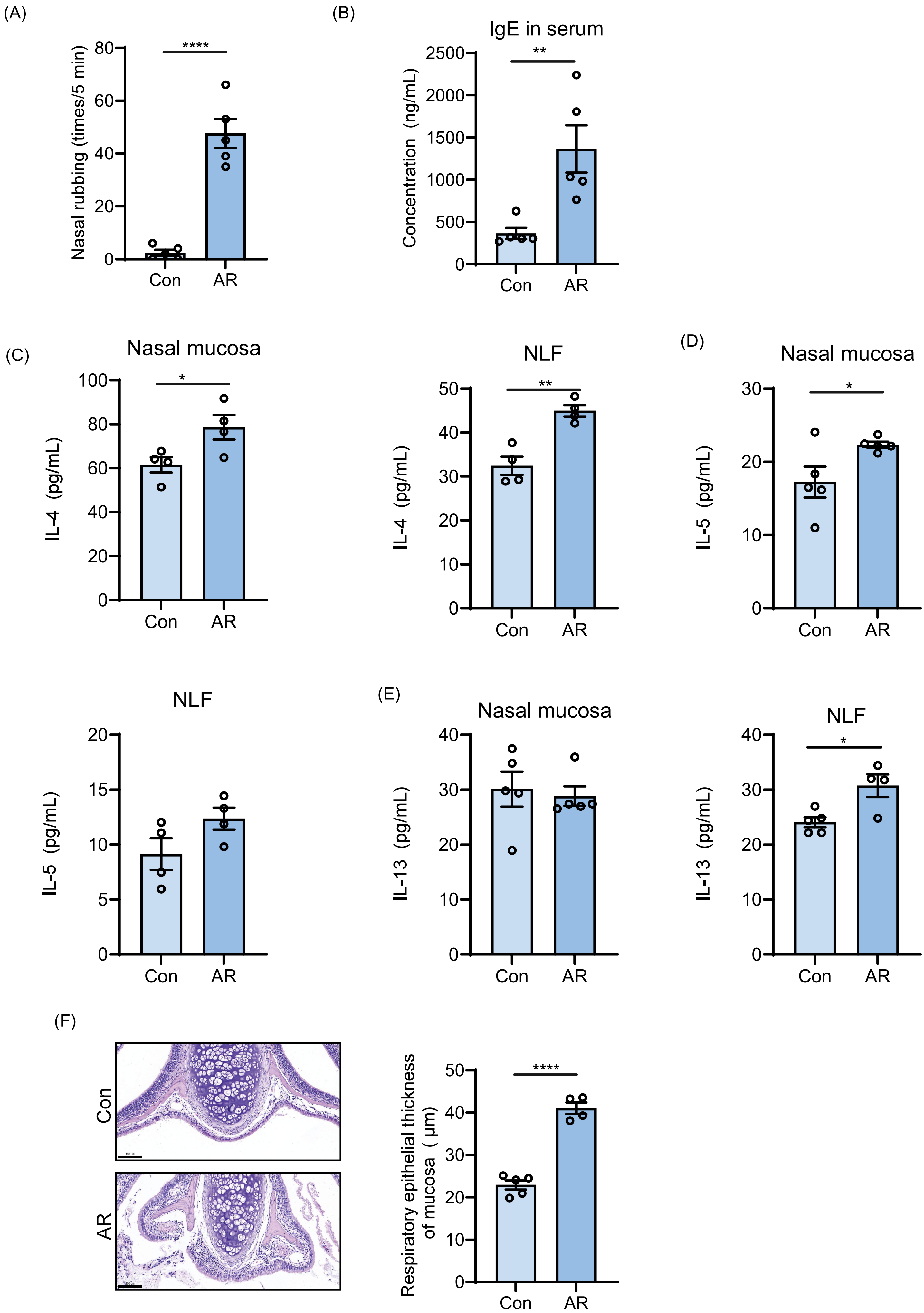

(A)
Nasal rubbing (times/5 min)
0
20
40
60
80
****
Con
AR
(B)
IgE in serum
Concentration (ng/mL)
0
500
1000
1500
2000
2500
**
Con
AR
(C)
Nasal mucosa
IL-4 (pg/mL)
0
20
40
60
80
100
*
Con
AR
NLF
IL-4 (pg/mL)
0
10
20
30
40
50
**
Con
AR
(D)
Nasal mucosa
IL-5 (pg/mL)
0
10
20
30
*
Con
AR
NLF
IL-5 (pg/mL)
0
5
10
15
20
Con
AR
(E)
Nasal mucosa
IL-13 (pg/mL)
0
10
20
30
40
Con
AR
NLF
IL-13 (pg/mL)
0
10
20
30
40
*
Con
AR
(F)
Con
AR
Respiratory epithelial thickness of mucosa (μm)
0
10
20
30
40
50
****
Con
AR
