# Supplementary material for: Staphylococcus aureus‐derived xanthine exacerbates allergic rhinitis‐induced olfactory dysfunction through promoting Th17 cell differentiation and IL‐17A production
Source: Clin Transl Immunology. 2026 Nov 1;15(11):e70127. doi: 10.1002/cti2.70127 (PMC13724795; doi:10.1002/cti2.70127)

(A) KEGG enrichment of all differential proteins

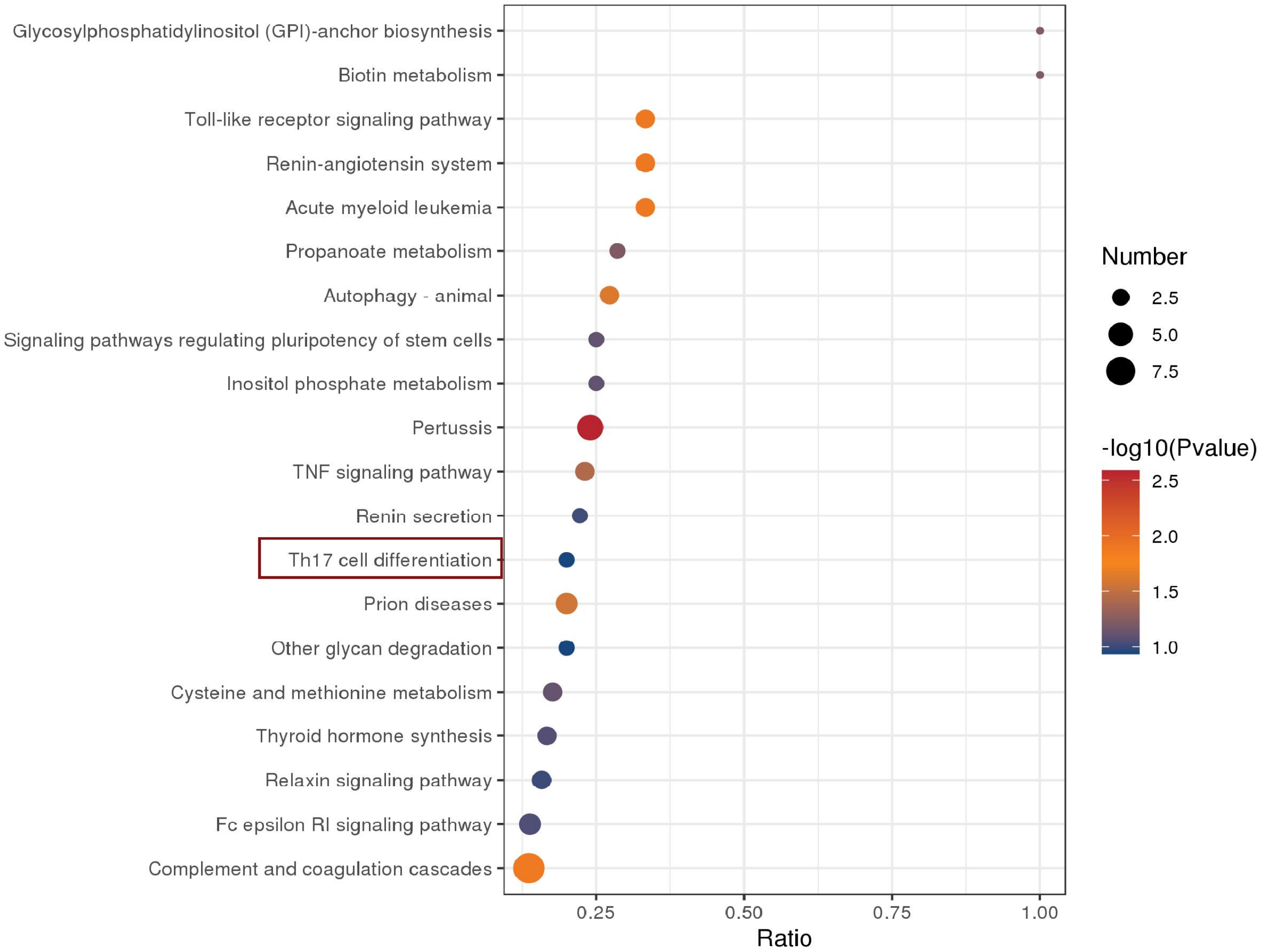

(B) KEGG enrichment of upregulated proteins

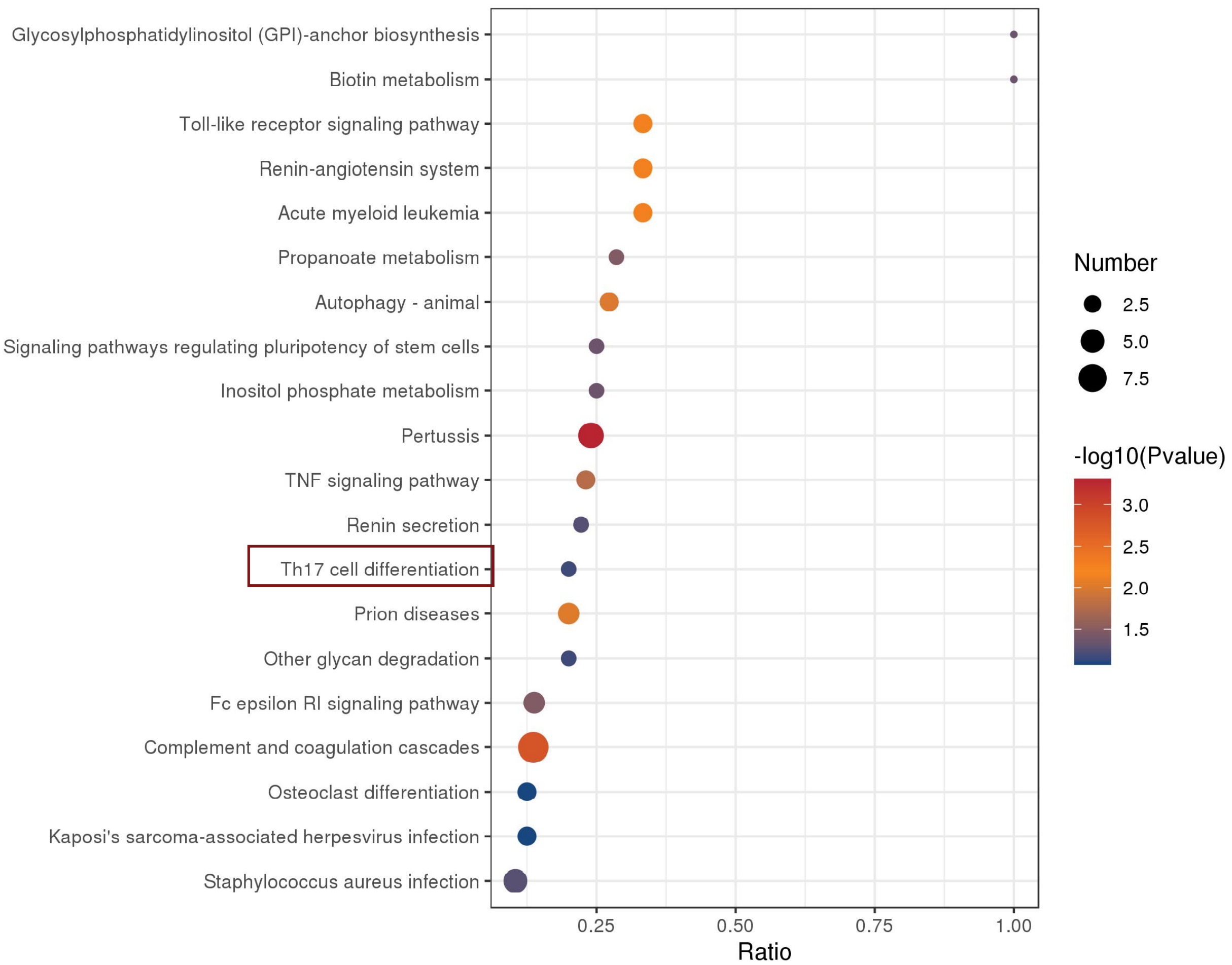

Supplement: Supplementary file 3 [file CTI2-15-e70127-s004.pdf]
