# Supplementary material for: Staphylococcus aureus‐derived xanthine exacerbates allergic rhinitis‐induced olfactory dysfunction through promoting Th17 cell differentiation and IL‐17A production
Source: Clin Transl Immunology. 2026 Nov 1;15(11):e70127. doi: 10.1002/cti2.70127 (PMC13724795; doi:10.1002/cti2.70127)
Supplement: Supplementary file 4 [file CTI2-15-e70127-s005.pdf]

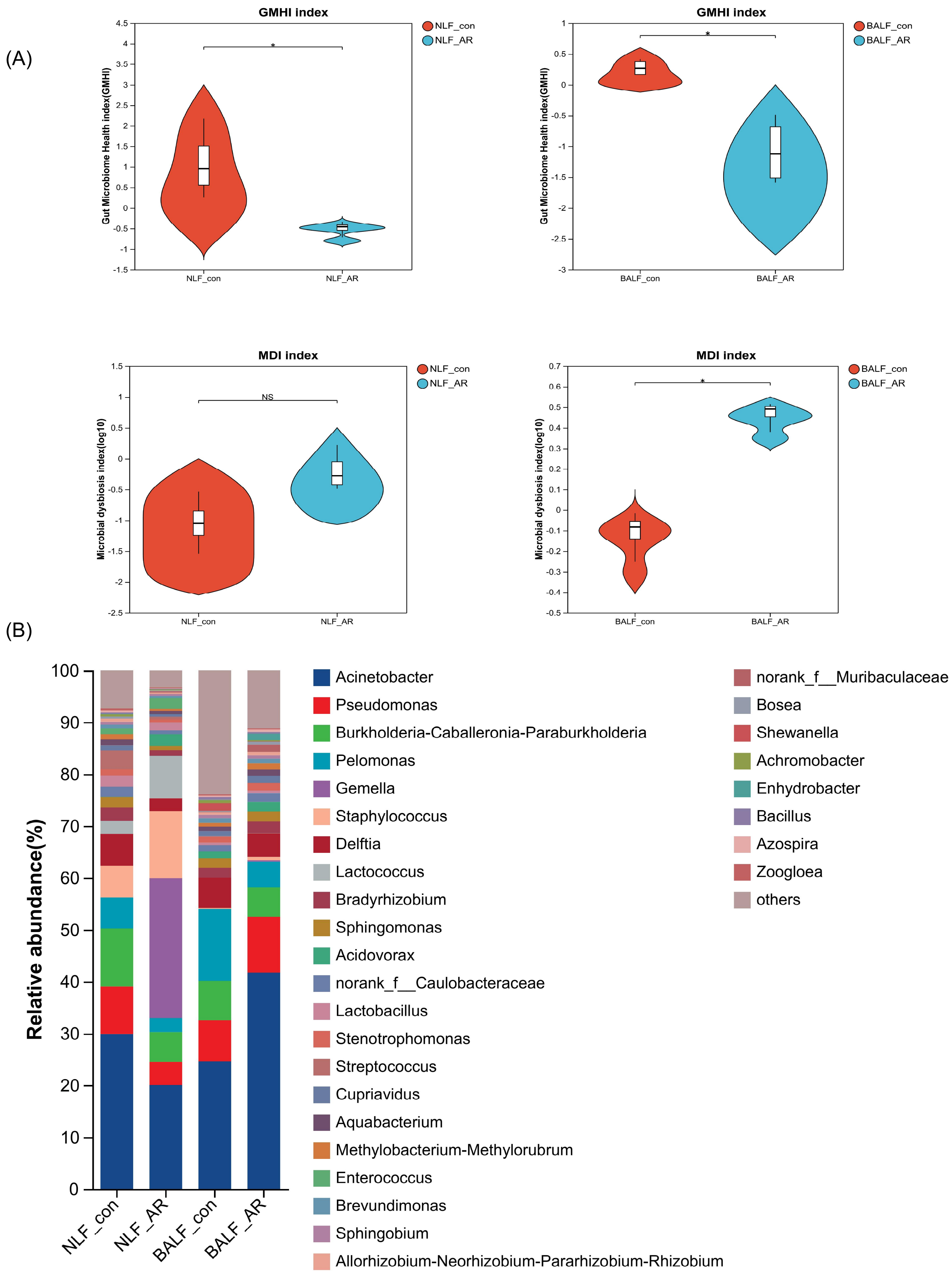
(A)
GMHI index
Gut Microbiome Health index(GMHI)
NLF_con
NLF_AR
*
GMHI index
Gut Microbiome Health index(GMHI)
BALF_con
BALF_AR
*
MDI index
Microbial dysbiosis index(log10)
NLF_con
NLF_AR
NS
MDI index
Microbial dysbiosis index(log10)
BALF_con
BALF_AR
*
(B)
Relative abundance(%)
NLF_con
NLF_AR
BALF_con
BALF_AR
Acinetobacter
Pseudomonas
Burkholderia-Caballeronia-Paraburkholderia
Pelomonas
Gemella
Staphylococcus
Delftia
Lactococcus
Bradyrhizobium
Sphingomonas
Acidovorax
norank_f__Caulobacteraceae
Lactobacillus
Stenotrophomonas
Streptococcus
Cupriavidus
Aquabacterium
Methylobacterium-Methylorubrum
Enterococcus
Brevundimonas
Sphingobium
Allorhizobium-Neorhizobium-Pararhizobium-Rhizobium
norank_f__Muribaculaceae
Bosea
Shewanella
Achromobacter
Enhydrobacter
Bacillus
Azospira
Zoogloea
others
