# Supplementary material for: Staphylococcus aureus‐derived xanthine exacerbates allergic rhinitis‐induced olfactory dysfunction through promoting Th17 cell differentiation and IL‐17A production
Source: Clin Transl Immunology. 2026 Nov 1;15(11):e70127. doi: 10.1002/cti2.70127 (PMC13724795; doi:10.1002/cti2.70127)
Supplement: Supplementary file 5 [file CTI2-15-e70127-s006.pdf]

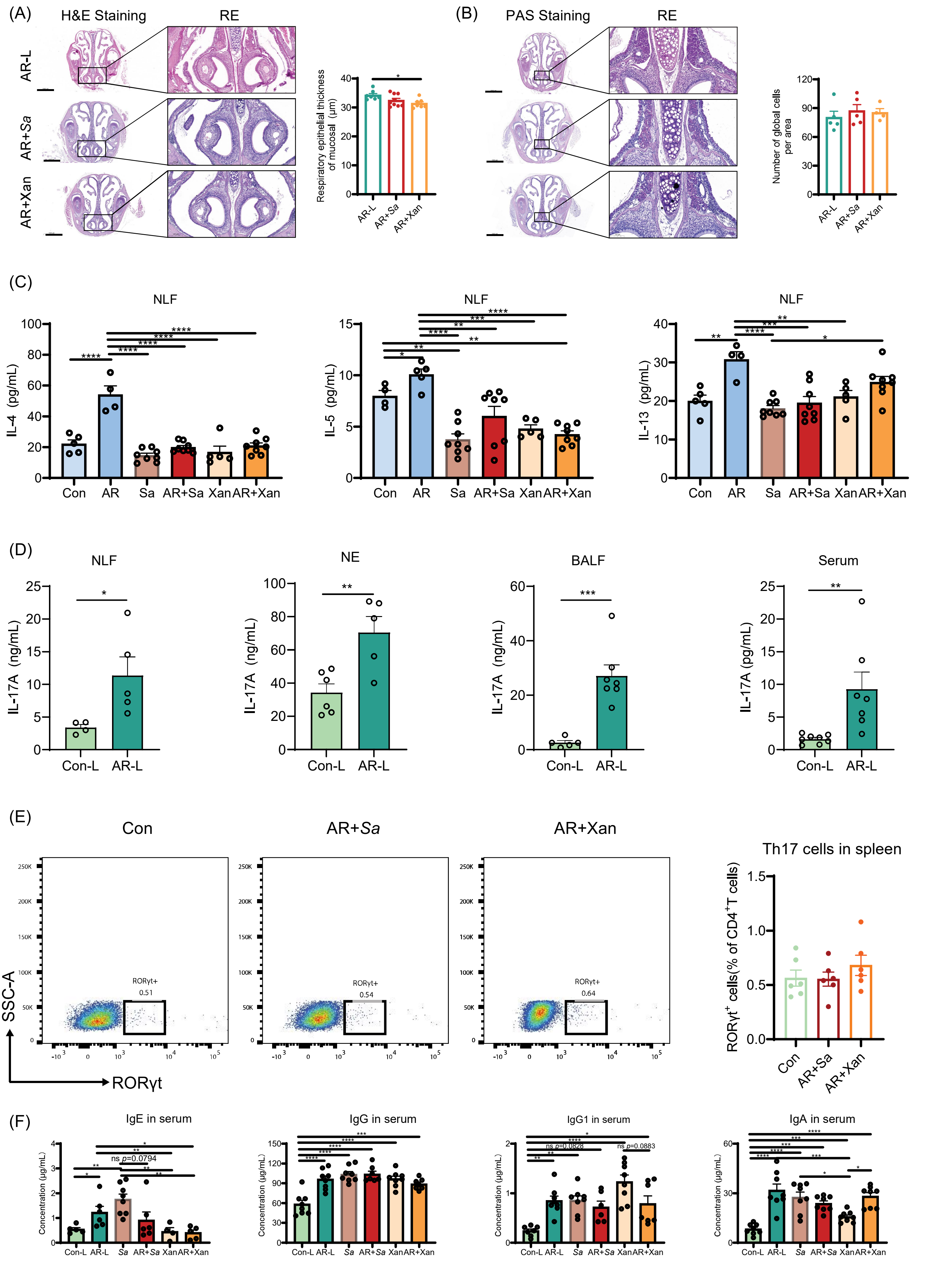

(A)
H&E Staining
RE
AR-L
AR+Sa
AR+Xan
Respiratory epithelial thickness of mucosal (μm)
(B)
PAS Staining
RE
Number of global cells per area
(C)
NLF
IL-4 (pg/mL)
IL-5 (pg/mL)
IL-13 (pg/mL)
Con
AR
Sa
AR+Sa
Xan
AR+Xan
(D)
NLF
NE
BALF
Serum
IL-17A (ng/mL)
IL-17A (pg/mL)
Con-L
AR-L
(E)
Con
AR+Sa
AR+Xan
RORγt+
0.51
0.54
0.64
SSC-A
RORγt
Th17 cells in spleen
RORγt+ cells(% of CD4+T cells)
(F)
IgE in serum
IgG in serum
IgG1 in serum
IgA in serum
Concentration (μg/mL)
ns p=0.0794
ns p=0.0828
ns p=0.0883
