# Supplementary material for: Staphylococcus aureus‐derived xanthine exacerbates allergic rhinitis‐induced olfactory dysfunction through promoting Th17 cell differentiation and IL‐17A production
Source: Clin Transl Immunology. 2026 Nov 1;15(11):e70127. doi: 10.1002/cti2.70127 (PMC13724795; doi:10.1002/cti2.70127)
Supplement: Supplementary file 6 [file CTI2-15-e70127-s007.pdf]

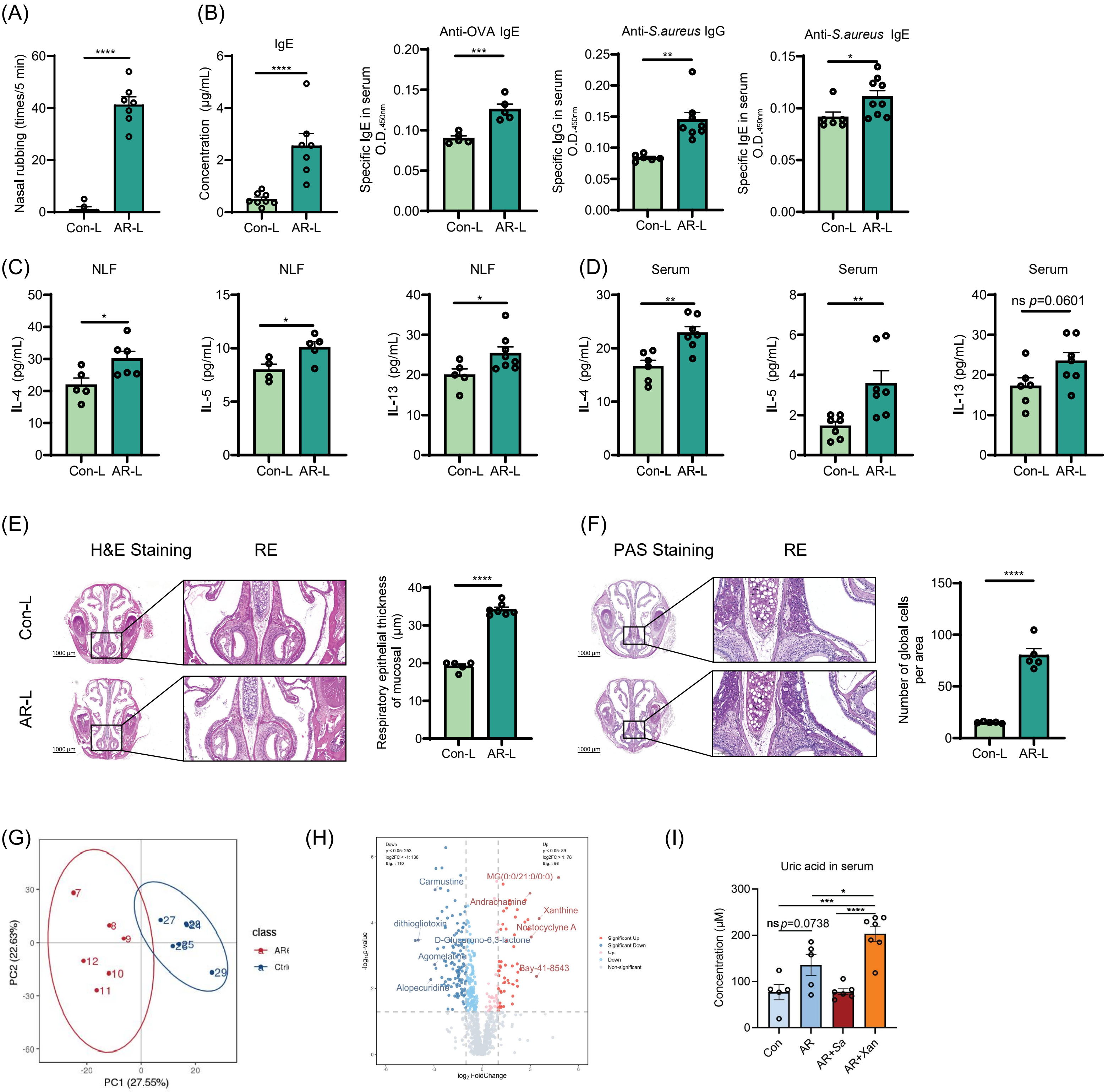
(A)
Nasal rubbing (times/5 min)
****
Con-L AR-L
(B)
IgE
Concentration (μg/mL)
****
Anti-OVA IgE
Specific IgE in serum O.D.450nm
***
Anti-S.aureus IgG
Specific IgG in serum O.D.450nm
**
Anti-S.aureus IgE
Specific IgE in serum O.D.450nm
*
(C)
NLF
IL-4 (pg/mL)
IL-5 (pg/mL)
IL-13 (pg/mL)
(D)
Serum
ns p=0.0601
(E)
H&E Staining
RE
Con-L
AR-L
Respiratory epithelial thickness of mucosal (μm)
(F)
PAS Staining
RE
Number of global cells per area
(G)
PC1 (27.55%)
PC2 (22.63%)
class
(H)
Carmustine
dithiogliotoxin
D-Glucurono-6,3-lactone
Agomelatine
Alopecuridine
MG(0:0/21:0/0:0)
Andrachamine
Xanthine
Nostocyclyne A
Bay-41-8543
(I)
Uric acid in serum
Concentration (μM)
ns p=0.0738
Con
AR
AR+Sa
AR+Xan
