# Supplementary material for: Staphylococcus aureus‐derived xanthine exacerbates allergic rhinitis‐induced olfactory dysfunction through promoting Th17 cell differentiation and IL‐17A production
Source: Clin Transl Immunology. 2026 Nov 1;15(11):e70127. doi: 10.1002/cti2.70127 (PMC13724795; doi:10.1002/cti2.70127)
Supplement: Supplementary file 7 [file CTI2-15-e70127-s008.docx]

**Inclusion and exclusion criteria for AR patients**

**Inclusion criteria**

(1) Age and Demographics: Aged between 18 and 65 years, strictly right-handed, and of native Han Chinese ethnicity;

(2) Clinical History: A typical AR symptoms persisting for > 2 years, in strict accordance with the Chinese Guidelines for Diagnosis and Treatment of Allergic Rhinitis (Revised 2022 Edition);

(3) Allergen Sensitization: A confirmed IgE-mediated sensitization to at least one common aeroallergen such as *Dermatophagoides farinae*, *Dermatophagoides pteronyssinus*, pollens, or animal dander). This was defined as a positive skin prick test (SPT, wheal diameter ≥ 3 mm larger than the negative control) and/or elevated serum specific IgE (sIgE) levels (≥ 0.35 IU/mL) determined by the ImmunoCAP system ;

(4) Informed consent: Voluntary participation with written informed consent obtained prior to any study-related procedures.

**Exclusion criteria**

(1) Rhinologic and Anatomical Factors: Concomitant chronic rhinosinusitis (CRS) with or without nasal polyps, structural nasal abnormalitiesClinically uncontrolled active infections (e.g., acute upper respiratory tract infections), or a history of nasal/sinus surgery;
(2) Systemic and Immunological Comorbidities: A history or presence of asthma , chronic obstructive pulmonary disease (COPD), autoimmune diseases, immunodeficiency disorders, or severe systemic diseases
(3) Medication Washout Requirements: Treatment with glucocorticoids, immunosuppressants within the past 4 weeks, treatment with antihistamines, leukotrienes, or decongestants within within the past 2 weeks; Current or previous history of allergen-specific immunotherapy (AIT).
(4) Special Populations & Protocol-Specific: Pregnant or breastfeeding.

**Inclusion and exclusion criteria for healthy subjects**

**Inclusion criteria**

(1) Negative objective testing: Negative results for all tested aeroallergens and food allergens on the skin prick test (SPT), and total serum IgE levels strictly within the normal physiological reference range.
(2) Demographic matching: Age- and sex-matched to the AR patients of native Han Chinese descent.

**Exclusion criteria**

The exclusion criteria for the healthy subjects are the same as those in the AR group.

**Supplementary Table1**

**Characteristics of subjects in the study**

|  | **HCs(n=20)** | **AR(n=22)** | ***P* value** |
| --- | --- | --- | --- |
| Age(y) | 37.9 ± 7.2 | 40.6± 10.1 | NS |
| Sex(%male/female) | 10.0/90.0 | 18.1/81.9 | NS |
| BMI(kg/m^2^) | 25.6±3.9 | 26.2±3.3 | NS |
| VAS | 9.4±0.8 | 7.0±1.1 | <0.001 |

Data are expressed as the mean±SEM. HCs = Healthy controls. AR= allergic rhinitis
